# Contribution of *CYP24A1* variants in coronary heart disease among the Chinese population

**DOI:** 10.1186/s12944-020-01356-x

**Published:** 2020-08-06

**Authors:** Peng Qian, Xuanchao Cao, Xianjing Xu, Mingqin Duan, Qian Zhang, Gairong Huang

**Affiliations:** Department of Geriatrics, Henan Provincial People’s Hospital, 7 weiwu road, Zhengzhou city, Henan province 450003 P. R. China

**Keywords:** Coronary heart disease, *CYP24A1*, Genetic polymorphisms, Case-control study, Stratified analysis, Multifactor dimensionality reduction, Chinese Han population

## Abstract

**Background:**

Cytochrome P450 (CYPs) participate in the mechanisms of cardiovascular disease. The purpose of this research was to evaluate the contributions of *CYP24A1* variants to coronary heart disease (CHD) among the Chinese Han population.

**Methods:**

This study included 505 CHD cases and 508 controls. Four variants of *CYP24A1* (rs2762934, rs1570669, rs6068816 and rs2296241) were chosen and genotyped by the Agena MassARRAY system among the Chinese population. The linkage between *CYP24A1* variants and CHD risk were assessed by logistic regression to compute the odds ratio (OR) and 95% confidence interval (CI). Then, multifactor dimensionality reduction (MDR) was applied to analyze the interactions of *CYP24A1* variants.

**Results:**

The results of this study showed that *CYP24A1* rs6068816 significantly enhanced CHD risk in multiple genetic models (allele: *P* = 0.014; codominant: *P* = 0.015; dominant: *P* = 0.043; recessive: *P* = 0.040; additive: *P* = 0.013), whereas rs2296241 was likely to protect individuals from CHD (codominant: *P* = 0.019; recessive: *P* = 0.013; additive: *P* = 0.033). Stratification analysis revealed that *CYP24A1* polymorphisms had strong relationships with CHD risk that were dependent on age, sex, Gensini grade and smoking status (*P* <  0.05). Moreover, a four-locus model (rs2762934, rs1570669, rs6068816 and rs2296241) had significant impact on CHD risk in MDR analysis.

**Conclusion:**

It revealed that *CYP24A1* variants were significantly linked with CHD susceptibility in the Chinese population.

## Introduction

Coronary heart disease (CHD) is a complex chronic inflammatory disease that is characterized by coronary artery remodeling and stenosis [1]. CHD is the leading cause of mortality and disability worldwide [2]. The World Health Organization reported that approximately 700,000 individuals die of CHD in China each year [3]. Previous studies have suggested that age, sex, diabetes and lifestyle factors (lack of exercise, smoking or alcohol use) are associated with susceptibility to CHD. CHD is a complex and heterogeneous illness that is attributed to the interaction of environmental and genetic factors, where the genetic factors are estimated to account for 30–60% of CHD risk [4, 5]. However, the role of genetic/environmental interactions in the development and progression of CHD requires further clarification.

Cytochrome P450 24 subfamily A member 1 (*CYP24A1*) encodes a 24-hydroxylase for degrading the active form of vitamin D through multiple pathways [6, 7]. The CYP450 proteins are monooxygenases that can catalyze reactions related to drug metabolism and lipid synthesis. It has been reported that the loss of *CYP24A1* function resulted in increased serum concentration of 1,25-dihydroxyvitamin D [7]. Previous studies revealed that vitamin D deficiency is a serious factor in the progression of cardiovascular disease [8–11]. In addition, *CYP24A1* polymorphisms were associated with many diseases, such as stroke, hypertension, hepatitis C virus infection and cancers. Wei Yang et al. reported that *CYP24A1* rs1570669 was linked to a reduced risk of stroke, and rs6068816 could increase susceptibility to ischemic stroke [12]. Five common variants of *CYP24A1* were reportedly related to cancer risks, including prostate, breast, colon and pancreatic cancers [13]. Nevertheless, the linkage between *CYP24A1* genetic variants and CHD risk in the Chinese population is not reported.

Considering the role of *CYP24A1* in multiple diseases, this study assumed that *CYP24A1* polymorphisms might be related to CHD risk. This study conducted a genetic association analysis of *CYP24A1* polymorphisms (rs2762934, rs1570669, rs6068816 and rs2296241) with CHD risk in the Chinese population.

## Methods

### Study subjects

This study included 505 patients with CHD and 508 age- and sex-matched controls. CHD patients were recruited from Yanan University Affiliated Hospital in China. CHD patients were diagnosed as having angiographically demonstrated stenosis (≥ 50%) in one or more major coronary arteries by two experienced interventional cardiologists. The heathy controls were also collected from the Healthy Center of Yanan University Affiliated Hospital. All controls were determined to be free of cardiovascular disease. Individuals with inflammatory diseases, cardiomyopathy, renal diseases (detected by hematuria tests) or other severe diseases were excluded from this study. Characteristics of the study subjects were collected by medical records and questionnaires, including age, sex, smoking and alcohol use, duration of CHD, complications, and levels of low-density lipoprotein (LDL), high-density lipoprotein (HDL), uric acid (UA), urea, platelet (PLT), white blood cells (WBC), red blood cells (RBC), hemoglobin (HGB), triglyceride (TG) and total cholesterol (TC). The study was performed in agreement with the Ethics Committee of Yanan University Affiliated Hospital, and written informed consent was obtained from study subjects.

### Single nucleotide polymorphisms (SNP) genotyping

According to the criteria of minor allele frequency (MAF) ≥ 0.05, four variants of *CYP24A1* (rs2762934, rs1570669, rs6068816 and rs2296241) were selected according to the HapMap database (http://www.hapmap.org). Then, blood samples were collected, and genomic DNA was extracted by a blood DNA kit (GoldMag Co. Ltd., Xi’an, China). Four variants were genotyped with the Agena MassARRAY system (Agena, San Diego, CA, USA). The primers of *CYP24A1* variants were designed by the Agena MassARRAY Assay Design 3.0 (San Diego, CA, USA; Supplementary Table 1). Agena Typer 4.0 Software (San Diego, CA, USA) was used for data management and analysis.

### Data analysis

SPSS 21.0 software (SPSS, Chicago, IL, USA) was applied to compute data, and the significance threshold was set at *P* <  0.05. The variables were compared by Student’s *t*-test and chi-square analysis, individually. Fisher’s exact test was used to evaluate the Hardy-Weinberg equilibrium (HWE) of each SNP in healthy controls. The relationship of *CYP24A1* polymorphisms and CHD susceptibility was assessed by logistic regression after adjustment for age and sex. This study determined the differences by an odds ratio (OR) with a 95% confidence interval (CI). Haplotype analysis of *CYP24A1* polymorphisms and CHD risk was further analyzed using Haploview and PLINK software. Multifactor dimensionality reduction (MDR, version 3.0.1) was conducted to assess the impact of *CYP24A1* polymorphisms on CHD susceptibility [14–16].

## Results

### Characteristics of the study population

Characteristics of the study individuals are shown in Table 1. This study enrolled 505 cases and 508 controls from China. The average ages of the two groups were 62.2 ± 10.4 and 61.5 ± 8.9 years old, respectively. The distributions of age and sex were similar between the case and control groups (age: *p* = 0.609; sex: *P* = 1.000). The levels of WBC, RBC, HGB, TG and TC in the two groups had significant differences (*P* <  0.05). Supplementary Table 2 did not show significant relationships between genotypes of *CYP24A1* variants and clinical characteristics of CHD cases (*P* > 0.05).

**Table 1:** Characteristics of the study population

Variables	Cases (***n*** = 505)	Controls (***n*** = 508)	*P*
Age, years	62.2 ± 10.4	61.5 ± 8.9	0.609
> 60	280 (55%)	286 (56%)	
≤ 60	225 (45%)	222 (44%)	
Sex			1.000
Male	334 (66%)	335 (66%)	
Female	171 (34%)	173 (34%)	
Smoking			
Yes	230 (46%)	112 (22%)	
No	185 (37%)	153 (30%)	
Drinking			
Yes	52 (10%)	109 (21%)	
No	303 (60%)	98 (19%)	
Duration, months			
≥ 40	99 (20%)		
< 40	232 (46%)		
Hypertension			
Yes	315 (62%)		
No	190 (38%)		
HDL (mmol/L)	1.10 ± 0.27	1.14 ± 0.23	0.115
LDL (mmol/L)	2.59 ± 0.84	2.60 ± 0.73	0.932
PLT (10^9^/L)	197.73 ± 59.62	207.30 ± 53.92	0.059
WBC	6.89 ± 2.17	5.71 ± 1.41	**< 0.001**
RBC	4.32 ± 0.61	4.73 ± 0.47	**< 0.001**
HGB	134.31 ± 19.54	144.26 ± 17.74	**< 0.001**
Urea	5.22 ± 2.25	7.50 ± 24.60	0.096
UA (μmol/L)	307.22 ± 92.67	318.73 ± 84.87	0.146
TG (mmol/L)	1.55 ± 0.90	1.81 ± 1.65	**0.022**
TC (mmol/L)	4.03 ± 0.96	5.39 ± 6.98	**0.001**

*HDL* high-density lipoprotein, *LDL* low-density lipoprotein, *PLT* platelet, *WBC* white blood cells, *RBC* red blood cells, *HGB* hemoglobin, *UA* uric acid, *TG* triglyceride, *TC* total cholesterol

Variables are presented as the mean ± SD

Bold-faced values indicate significant difference (*P* <  0.05)

### Association of *CYP24A1* polymorphisms and CHD risk

Four SNPs were genotyped in two groups, and all SNPs in the control group were HWE compliant (HWE *P* > 0.05, Table 2). For *CYP24A1* rs6068816, the frequency distribution of the T allele was higher in CHD patients than that in heathy controls (*P* = 0.014). HaploReg showed that *CYP24A1* polymorphisms were regulated by Enhancer histone marks, DNAse, motifs, proteins bound, motifs changed, NHGRI/EBI GWAS hits, SiPhy cons and Selected eQTL hits. Table 3 revealed that rs6068816 significantly increased CHD susceptibility in codominant (OR = 1.64, 95% CI = 1.10–2.46, *P* = 0.015), dominant (OR = 1.30, 95% CI = 1.01–1.67, *P* = 0.043), recessive (OR = 1.49, 95% CI = 1.02–2.17, *P* = 0.040) and additive (OR = 1.26, 95% CI = 1.05–1.52, *P* = 0.013) models. *CYP24A1* rs2296241 had a strong linkage with lower susceptibility to CHD (codominant: OR = 0.63, 95% CI = 0.43–0.93, *P* = 0.019; recessive: OR = 0.66, 95% CI = 0.47–0.92, *P* = 0.013 and additive: OR = 0.82, 95% CI = 0.68–0.98, *P* = 0.033).

**Table 2:** Allele frequency of *CYP24A1* SNPs and their associations with risk of CHD

SNP	Genotype	Location	Cases	Controls	MAF-Case	MAF-Control	HWE ***P***	OR(95%CI)	***P***	HaploReg
rs2762934	A/G	3′-UTR	116/890	108/908	0.115	0.106	1.000	1.10 (0.83–1.45)	0.519	Enhancer histone marks, DNAse, Motifs, Proteins bound, Motifs changed
rs1570669	A/G	Intronic	379/629	401/615	0.376	0.395	0.403	0.92 (0.77–1.11)	0.388	DNAse, Proteins bound, Motifs changed, NHGRI/EBI GWAS hits
rs6068816	T/C	Synonymous	380/622	330/680	0.379	0.327	0.920	1.26 (1.05–1.51)	**0.014**	SiPhy cons, DNAse, Proteins bound, Motifs changed,
rs2296241	A/G	Synonymous	423/587	469/547	0.419	0.462	0.212	0.84 (0.71–1.00)	0.052	SiPhy cons, Enhancer histone marks, DNAse, Proteins bound, Motifs changed, Selected eQTL hits

*SNP* single nucleotide polymorphism, *CHD* coronary heart disease, *MAF* minor allele frequency, *OR* odds ratio, *95% CI* 95% confidence interval

Bold-faced values indicate significant difference (*P* <  0.05)

**Table 3:** Genotypes frequencies of *CYP24A1* SNPs and their associations with risk of CHD

SNP	Genotype	Cases	Controls	Without adjustment		With adjustment	
				OR(95%CI)	*P*^**a**^	OR(95%CI)	*P*^**b**^
rs2762934							
co-dominant	AA	4	5	0.83 (0.22–3.11)	0.781	0.80 (0.21–3.02)	0.745
	GA	108	98	1.14 (0.84–1.55)	0.398	1.13 (0.83–1.54)	0.436
	GG	391	405	1		1	
dominant	AA-AG	112	103	1.13 (0.83–1.52)	0.439	1.11 (0.82–1.51)	0.484
	GG	391	405	1		1	
recessive	AA	4	5	0.81 (0.22–3.02)	0.750	0.78 (0.21–2.94)	0.716
	AG-GG	499	503	1		1	
additive				1.1 (0.83–1.46)	0.512	1.09 (0.82–1.44)	0.563
rs1570669							
co-dominant	AA	75	74	0.92 (0.63–1.34)	0.655	0.91 (0.62–1.33)	0.637
	GA	229	253	0.82 (0.63–1.07)	0.146	0.82 (0.63–1.07)	0.150
	GG	200	181	1		1	
dominant	AA-AG	304	327	0.84 (0.65–1.09)	0.184	0.84 (0.65–1.09)	0.184
	GG	200	181	1		1	
recessive	AA	75	74	1.03 (0.72–1.45)	0.888	1.02 (0.72–1.44)	0.914
	AG-GG	429	434	1		1	
additive				0.92 (0.77–1.11)	0.386	0.92 (0.77–1.10)	0.378
rs6068816							
co-dominant	TT	74	53	1.63 (1.09–2.44)	**0.017**	1.64 (1.10–2.46)	**0.015**
	TC	232	224	1.21 (0.93–1.58)	0.157	1.21 (0.93–1.58)	0.152
	CC	195	228	1		1	
dominant	TT-TC	306	277	1.29 (1.01–1.66)	**0.046**	1.30 (1.01–1.67)	**0.043**
	CC	195	228	1		1	
recessive	TT	74	53	1.48 (1.01–2.15)	**0.042**	1.49 (1.02–2.17)	**0.040**
	TC-CC	427	452	1		1	
additive				1.26 (1.05–1.51)	**0.014**	1.26 (1.05–1.52)	**0.013**
rs2296241							
co-dominant	AA	71	101	0.64 (0.44–0.94)	**0.023**	0.63 (0.43–0.93)	**0.019**
	GA	281	267	0.96 (0.73–1.28)	0.795	0.95 (0.71–1.26)	0.702
	GG	153	140	1		1	
dominant	AA-AG	352	368	0.88 (0.67–1.15)	0.337	0.86 (0.65–1.13)	0.280
	GG	153	140	1		1	
recessive	AA	71	101	0.66 (0.47–0.92)	**0.014**	0.66 (0.47–0.92)	**0.013**
	AG-GG	434	407	1		1	
additive				0.82 (0.68–0.99)	**0.041**	0.82 (0.68–0.98)	**0.033**

*SNP* single nucleotide polymorphism, *CHD* coronary heart disease, *OR* odds ratio, *95% CI* 95% confidence interval

*P*^a^ values were calculated by logistic regression analysis with the comparison between CHD patients and healthy controls

*P*^b^ values were calculated by logistic regression analysis with adjustment for age and gender

Bold-faced values indicate significant difference (*P* <  0.05)

Furthermore, stratification analysis of *CYP24A1* polymorphisms with CHD risk was performed (Table 4). In the subgroup of age ≤ 60, rs2762934 and rs6068816 significantly increased CHD risk (*P* <  0.05). *CYP24A1* rs6068816 was also linked with higher susceptibility to CHD in the subgroup of men (homozygote: OR = 2.03, 95% CI = 1.21–3.40, *P* = 0.007; dominant: OR = 1.42, 95% CI = 1.04–1.93, *P* = 0.028; recessive: OR = 1.77, 95% CI = 1.09–2.88, *P* = 0.022; additive: OR = 1.38, 95% CI = 1.10–1.74, *P* = 0.006; allele: OR = 1.37, 95% CI = 1.09–1.72, *P* = 0.007) and smokers (homozygote: OR = 3.02, 95% CI = 1.31–6.99, *P* = 0.010; recessive: OR = 2.60, 95% CI = 1.17–5.78, *P* = 0.019; additive: OR = 1.57, 95% CI = 1.11–2.23, *P* = 0.011; allele: OR = 1.61, 95% CI = 1.15–2.27, *P* = 0.006). Rs1570669 and rs2296241 had strong relationships with CHD susceptibility in Gensini grade and male subgroups.

**Table 4:** Stratification analyses of the association of *CYP24A1* polymorphisms with susceptibility of CHD

Polymorphisms	Subgroups	Homozygote		Heterozygote		Dominant		Recessive		Additive		Allele	
		OR (95%CI)	*P*	OR (95%CI)	*P*	OR (95%CI)	*P*	OR (95%CI)	*P*	OR (95%CI)	*P*	OR (95%CI)	*P*
rs2762934	Age (≤ 60)	–	–	1.51 (0.93–2.44)	0.090	1.57 (0.97–2.53)	0.060	–	–	1.61 (1.01–2.56)	**0.040**	1.57 (1.00–2.46)	**0.048**
rs1570669	Gensini grade	0.61 (0.31–1.20)	0.156	1.79 (1.06–3.02)	**0.031**	1.33 (0.82–2.14)	0.243	1.45 (0.24–0.85)	**0.013**	0.92 (0.66–1.29)	0.637	0.90 (0.64–1.26)	0.544
rs6068816	Age (≤ 60)	2.76 (1.47–5.20)	**< 0.001**	1.27 (0.85–1.90)	0.250	1.49 (1.01–2.18)	**0.040**	2.42 (1.34–4.38)	**< 0.001**	1.53 (1.15–2.03)	**< 0.001**	1.51 (1.15–1.99)	**0.003**
	Men	2.03 (1.21–3.40)	**0.007**	1.30 (0.94–1.80)	0.116	1.42 (1.04–1.93)	**0.028**	1.77 (1.09–2.88)	**0.022**	1.38 (1.10–1.74)	**0.006**	1.37 (1.09–1.72)	**0.007**
	Smoker	3.02 (1.31–6.99)	**0.010**	1.33 (0.82–2.16)	0.251	1.56 (0.98–2.49)	0.060	2.60 (1.17–5.78)	**0.019**	1.57 (1.11–2.23)	**0.011**	1.61 (1.15–2.27)	**0.006**
rs2296241	Men	0.60 (0.37–0.96)	**0.031**	0.77 (0.54–1.09)	0.140	0.73 (0.52–1.01)	0.059	0.71 (0.47–1.07)	0.099	0.77 (0.61–0.97)	**0.027**	1.25 (0.16–1.90)	0.180

*OR* Odds ratio, *CI* Confidence interval, *CHD* coronary heart disease

Bold-faced values indicate significant difference (*P* < 0.05)

### Haplotype and MDR analysis

**Fig. 1:**
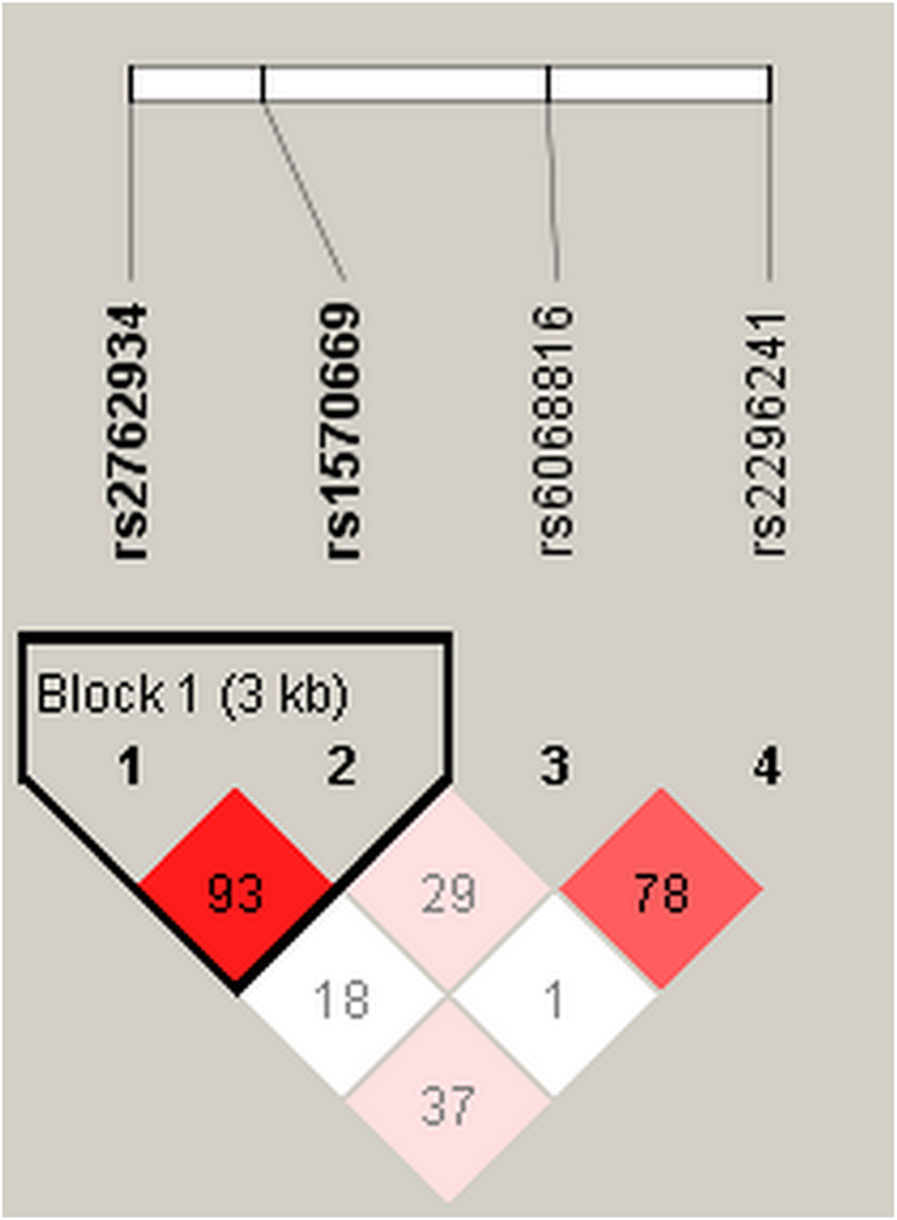
Linkage disequilibrium (LD) plots containing two polymorphisms from *CYP24A1*. Block 1 includes rs2762934 and rs1570669. The numbers inside the diamonds indicate the D’ for pairwise analyses

The haplotype analysis of *CYP24A1* polymorphisms and CHD risk was performed, and there was no significant linkage between haplotypes and susceptibility to CHD (*P* > 0.05). One block (rs2762934 and rs1570669) was presented in Fig. 1. In addition, the effects of SNP-SNP interactions among four SNPs in *CYP24A1* are shown in Table 5. MDR analysis showed that a four-locus model, including rs2762934, rs1570669, rs6068816 and rs2296241, was the best model (cross-validation consistency = 10/10, accuracy = 0.580, *P* <  0.001).

**Table 5:** MDR analysis of SNP-SNP interactions

Model	Training Bal. Acc.	Testing Bal. Acc.	CV Consistency	Accuracy	Sensitivity	Specificity	OR(95%CI)	*P*
rs2296241	0.533	0.533	10/10	0.533	0.614	0.452	1.31 (1.02–1.68)	**0.035**
rs1570669,rs2296241	0.550	0.548	10/10	0.550	0.721	0.378	1.57 (1.20–2.05)	**0.001**
rs1570669,rs6068816,rs2296241	0.567	0.509	6/10	0.565	0.457	0.673	1.74 (1.35–2.24)	**< 0.001**
rs2762934,rs1570669,rs6068816,rs2296241	0.586	0.484	10/10	0.580	0.659	0.501	1.94 (1.51–2.50)	**< 0.001**

*MDR* multifactor dimensionality reduction, *SNP* single nucleotide polymorphism, *CV* cross-validation, *OR* odds ratio, *CI* confidence interval

Bold-faced values indicate significant difference (*P* < 0.05)

## Discussion

This study investigated the relationship of four *CYP24A1* SNPs (rs2762934, rs1570669, rs6068816 and rs2296241) on CHD risk; rs6068816 and rs2296241 indicated susceptibility to CHD in the Chinese Han population (*P* <  0.05). Subgroup analysis demonstrated that rs2762934 enhanced CHD risk among younger individuals (age ≤ 60), rs2296241 decreased the risk of CHD among men, and rs6068816 was significantly linked with a higher risk of CHD in the subgroups of age ≤ 60, men, and smokers. For CHD patients, rs1570669 could enhance CHD risk in the subgroup of Gensini grade. It also showed one block (rs2762934 and rs1570669). These results might provide a new insight on the contribution of *CYP24A1* polymorphisms in CHD risk among the Chinese population.

Vitamin D is a soluble steroid hormone that plays an important role in calcium homeostasis, skeletal health and cardiovascular pathophysiology [2]. It has been reported that low levels of vitamin D are linked with a variety of diseases, including diabetes, autoimmune disorders, skin diseases, cardiovascular diseases and cancers [17–19]. Vitamin D is produced in the skin and is metabolized in the liver and kidney, which requires CYP450 enzymes, such as CYP2R1, CYP27B1, CYP24A1 [20]. Previous studies indicated that rs6068816 affected cancer risk through the vitamin D pathway [21, 22] and that rs2296241 was related to vitamin D deficiency in the development of food sensitization [23]. The study mainly focused on the linkage of *CYP24A1* and CHD among the Chinese Han population. The results showed *CYP24A1* polymorphisms were associated with CHD susceptibility. *CYP24A1* rs6068816 significantly increased the risk of CHD, whereas no significant linkage of hypertension with rs6068816 and no difference in vitamin D levels among the genotypes of rs6068816 were found [24]. Moreover, this study suggested that rs2296241 could protect individuals from CHD. Lu et al. also reported that rs2296241 had strong associations with systolic blood pressure (BP), diastolic BP, pulse pressure, or mean arterial pressure in the Women’s Genome Healthy Study [25]. These findings suggests that *CYP24A1* polymorphisms may participate in the progression of CHD, and this is likely due to the effects of *CYP24A1* on regulating the levels of vitamin D. Further studies are required to verify these results.

There are differences between the influence of age and sex on CHD; CHD more frequently occurs in men (17.6%) than women (10.6%) [26]. The mortality of CHD is also different in adults between the two sexes, and it increases with age [27]. However, the causes of age and sex differences in CHD are still unclear. Hence, this study evaluated the influences of *CYP24A1* polymorphisms on CHD risk in the subgroups of age and sex. The impact of rs2762934, rs6068816 and rs2296241 on CHD risk varied with age and sex. In addition, smoking was a major risk factor for CHD [28]. The results showed that *CYP24A1* rs6068816 significantly increased the risk of CHD among men, smokers and subjects aged 60 years old or younger. A meta-analysis involving 20,593 cases and 25,458 controls revealed that there were no associations of rs6068816 with overall cancer risks [13], but Wei Yang el al. reported that rs6068816 could enhance the susceptibility of ischemic stroke in the Chinese population [12]. These studies would provide insight on the diagnosis, prevention or treatment of cardiovascular disease. Last, the study divided CHD patients into different groups according to Gensini grade, and *CYP24A1* rs1570669 could worsen the condition of patients. This finding gives us a clue as to individual treatments for CHD patients.

### Study strength and limitations

The strengths of this study were listed as following. First, the study reported the linkage of *CYP24A1* polymorphisms and CHD risk, and these impacts were related to multiple factors. Second, the association of genetic polymorphisms with CHD susceptibility was also assessed by many subgroups, as well as haplotype and MDR analysis. Third, this study used clinical data from a study population of 1013 individuals. Finally, the study provides a new candidate gene or variants for studying the subsequent pathogenesis of CHD. These findings may facilitate the diagnosis and prevention of CHD in the future.

There are some deficiencies in this study, which should be listed. First, sample size was relatively small, such that it could not give enough statistical power. Additionally, a selection bias may exist in this case-control study. Third, more risk indicators were not analyzed in the study due to limitations of information. Fourth, the study did not assay vitamin D levels in cases and controls. Finally, more studies should be performed to validate these results.

## Conclusions

The study revealed that *CYP24A1* variants were nominally linked with CHD susceptibility, and the impacts of *CYP24A1* polymorphisms on CHD risk were related to age, sex, Gensini grade or smoking status. It suggested that *CYP24A1* variants might take part in the development of CHD. It provides a scientific basis for the underlying mechanism of CHD. In the future, these findings will guide personalized medicine for the treatment of CHD.

## Supplementary information

**Additional file 1: Supplementary Table 1** Primers of *CYP24A1* polymorphisms**. Supplementary Table 2** Clinical characteristics of CHD patients based on *CYP24A1* polymorphisms.

## Data Availability

All data generated or analyzed during this study are included in this published article.

## Abbreviations

CHD: Coronary heart disease
CYP24A1: Cytochrome P450 24 subfamily A member 1
HDL: High-density lipoprotein
LDL: Low-density lipoprotein
UA: Uric acid
PLT: Platelet
WBC: White blood cells
RBC: Red blood cells
HGB: Hemoglobin
TG: Triglyceride
TC: Total cholesterol
MAF: Minor allele frequency
HWE: Hardy-Weinberg equilibrium
OR: Odds ratio
CI: 95% confidence interval
MDR: Multifactor dimensionality reduction
BP: Blood pressure
