# Supplementary material for: Contribution of CYP24A1 variants in coronary heart disease among the Chinese population
Source: Lipids Health Dis. 2020 Aug 6;19:181. doi: 10.1186/s12944-020-01356-x (PMC7412795; doi:10.1186/s12944-020-01356-x)
Supplement: Supplementary file 1 — Additional file 1: Supplementary Table 1 Primers of CYP24A1 polymorphisms. Supplementary Table 2 Clinical characteristics of CHD patients based on CYP24A1 polymorphisms. [file 12944_2020_1356_MOESM1_ESM.docx]

**Supplementary Table 1** Primers of *CYP24A1* polymorphisms

| **SNP** | **1st-PCRP** | **2nd-PCRP** | **UEP_DIR** | **UEP_SEQ** |
| --- | --- | --- | --- | --- |
| rs2762934 | ACGTTGGATGGTTCCAGAAGCTGTACTGTC | ACGTTGGATGTGTAGAATGCCTTGGATCCC | F | CCCAGCACTCAGTCC |
| rs1570669 | ACGTTGGATGAGACGAAGTTGAGGCTCACA | ACGTTGGATGGAATTCACGGCTATGGAGAC | R | cctcGACCTGCATTCAGTTTCA |
| rs6068816 | ACGTTGGATGCTTCCAGAACGAACATTGTC | ACGTTGGATGCGACTGGAGTGACCATCATC | F | ccctcCCATCATCCTCCCAAA |
| rs2296241 | ACGTTGGATGAAATGTGTCTTTTGCGGTTG | ACGTTGGATGTCTTCAACGTGGCCTCTTTC | F | TCATCTATTCTGCCCATAAAATC |

**Supplementary Table 2** Clinical characteristics of CHD patients based on *CYP24A1* polymorphisms

| **Characteristics** | **rs2762934** | | | |  | **rs1570669** | | | |
| --- | --- | --- | --- | --- | --- | --- | --- | --- | --- |
|  | **AA** | **AG** | **GG** | *P* |  | **AA** | **AG** | **GG** | *P* |
| HDL (mmol/L) | 0.94 ± 0.01 | 1.11 ± 0.29 | 1.10 ± 0.26 | 0.075 |  | 1.13 ± 0.30 | 1.09 ± 0.24 | 1.10 ± 0.28 | 0.074 |
| LDL (mmol/L) | 1.95 ± 0.01 | 2.68 ± 0.91 | 2.57 ± 0.83 | 0.086 |  | 2.71 ± 0.95 | 2.58 ± 0.85 | 2.56 ± 0.79 | 0.181 |
| PLT (109/L) | 219.08 ± 71.01 | 191.40 ± 58.86 | 198.85 ± 59.88 | 0.888 |  | 208.22 ± 61.92 | 195.98 ± 60.61 | 195.88 ± 57.55 | 0.708 |
| WBC | 9.98 ± 0.11 | 6.81 ± 2.08 | 6.87 ± 2.19 | 0.220 |  | 6.67 ± 2.02 | 6.95 ± 2.36 | 6.90 ± 2.00 | 0.620 |
| RBC | 4.43 ± 0.42 | 4.30 ± 0.59 | 4.32 ± 0.62 | 0.886 |  | 4.34 ± 0.56 | 4.30 ± 0.65 | 4.33 ± 0.59 | 0.650 |
| HGB | 137.67 ± 6.35 | 132.86 ± 17.15 | 134.80 ± 20.15 | 0.554 |  | 133.59 ± 17.07 | 133.68 ± 17.86 | 135.36 ± 22.28 | 0.994 |
| Urea | 4.26 ± 1.90 | 5.06 ± 1.77 | 5.28 ± 2.37 | 0.995 |  | 4.96 ± 1.54 | 5.45 ± 2.79 | 5.05 ± 1.61 | 0.093 |
| Uric acid (μmol/L) | 329.27 ± 92.90 | 307.92 ± 94.48 | 306.52 ± 92.53 | 0.928 |  | 287.86 ± 71.80 | 314.70 ± 95.57 | 305.52 ± 95.73 | 0.104 |
| TG (mmol/L) | 2.06 ± 0.01 | 1.56 ± 0.70 | 1.55 ± 0.95 | 0.325 |  | 1.55 ± 0.62 | 1.59 ± 0.99 | 1.52 ± 0.86 | 0.512 |
| TC (mmol/L) | 3.30 ± 0.01 | 4.15 ± 1.03 | 4.00 ± 0.95 | 0.159 |  | 4.18 ± 1.03 | 4.02 ± 0.97 | 3.98 ± 0.93 | 0.825 |
| **Characteristics** | **rs6068816** | | | |  | **rs2296241** | | | |
|  | **TT** | **CT** | **CC** | *P* |  | **AA** | **AG** | **GG** | *P* |
| HDL (mmol/L) | 1.11 ± 0.26 | 1.13 ± 0.27 | 1.05 ± 0.27 | 0.749 |  | 1.03 ± 0.26 | 1.13 ± 0.28 | 1.07 ± 0.23 | 0.179 |
| LDL (mmol/L) | 2.64 ± 0.80 | 2.63 ± 0.86 | 2.53 ± 0.83 | 0.812 |  | 2.56 ± 0.90 | 2.62 ± 0.72 | 2.57 ± 0.77 | 0.472 |
| PLT (109/L) | 207.77 ± 65.87 | 199.59 ± 58.26 | 191.31 ± 57.82 | 0.550 |  | 205.16 ± 58.82 | 194.19 ± 60.88 | 200.06 ± 57.98 | 0.942 |
| WBC | 7.31 ± 1.92 | 7.04 ± 2.43 | 6.50 ± 1.87 | 0.128 |  | 6.64 ± 1.85 | 6.73 ± 2.11 | 7.26 ± 2.37 | 0.448 |
| RBC | 4.38 ± 0.49 | 4.33 ± 0.60 | 4.26 ± 0.67 | 0.693 |  | 4.30 ± 0.66 | 4.27 ± 0.66 | 4.40 ± 0.49 | 0.208 |
| HGB | 134.45 ± 15.95 | 134.00 ± 16.61 | 134.81 ± 23.94 | 0.569 |  | 134.49 ± 19.96 | 133.15 ± 16.98 | 136.14 ± 23.04 | 0.704 |
| Urea | 5.33 ± 1.63 | 5.28 ± 2.66 | 5.09 ± 1.92 | 0.850 |  | 4.58 ± 1.43 | 5.14 ± 1.94 | 5.68 ± 2.88 | 0.176 |
| Uric acid (μmol/L) | 312.17 ± 91.41 | 297.63 ± 95.20 | 317.44 ± 89.87 | 0.629 |  | 314.07 ± 100.41 | 300.06 ± 94.08 | 315.90 ± 86.12 | 0.750 |
| TG (mmol/L) | 1.78 ± 1.10 | 1.49 ± 0.91 | 1.55 ± 0.76 | 0.071 |  | 1.60 ± 0.73 | 1.47 ± 0.78 | 1.67 ± 1.12 | 0.153 |
| TC (mmol/L) | 4.14 ± 0.92 | 4.09 ± 1.01 | 3.92 ± 0.92 | 0.864 |  | 3.93 ± 1.02 | 4.07 ± 1.00 | 4.00 ± 0.88 | 0.350 |

HDL, high-density lipoprotein; LDL, low-density lipoprotein; PLT, platelet; WBC, white blood cells; RBC, red blood cells; HGB, hemoglobin; UA, uric acid; TG, triglyceride; TC, total cholesterol

Bold-faced values indicate significant difference (*P* < 0.05)
